# Culture and biology in the origins of linguistic structure

**DOI:** 10.3758/s13423-016-1166-7

**Published:** 2017-01-24

**Authors:** Simon Kirby

**Affiliations:** Centre for Language Evolution, University of Edinburgh, Edinburgh, UK

**Keywords:** Language evolution, Cultural evolution, Computational modeling, Iterated learning

## Abstract

Language is systematically structured at all levels of description, arguably setting it apart from all other instances of communication in nature. In this article, I survey work over the last 20 years that emphasises the contributions of individual learning, cultural transmission, and biological evolution to explaining the structural design features of language. These 3 complex adaptive systems exist in a network of interactions: individual learning biases shape the dynamics of cultural evolution; universal features of linguistic structure arise from this cultural process and form the ultimate linguistic phenotype; the nature of this phenotype affects the fitness landscape for the biological evolution of the language faculty; and in turn this determines individuals’ learning bias. Using a combination of computational simulation, laboratory experiments, and comparison with real-world cases of language emergence, I show that linguistic structure emerges as a natural outcome of cultural evolution once certain minimal biological requirements are in place.

## Introduction

There is undoubtedly something unusual about humans. Our species currently numbers around 7 billion, whereas there are fewer than 300,000 remaining of our nearest living relatives, the chimpanzees. We have expanded across the planet to inhabit nearly all the available habitats, and have even now taken up permanent residence off world with over 15 years of continuous habitation of space. By any reasonable measure, we are a spectacularly successful species. But what is it about us that makes us such an extraordinary primate? An obvious candidate is the fact that we are able to share and accumulate knowledge. Our understanding of the world, and consequently our ability to shape that world, is not limited to the knowledge and skills which we can acquire in our own lifetimes. This trait, which biologists Eors Szathmary and John Maynard Smith called unlimited heredity (Maynard Smith & Szathmary, 1995), is uniquely human and is enabled by another unique trait: language.

Despite the ubiquity of communication in nature, language is strikingly different in its structure from other forms of communication, and it is this difference in structure that enables unlimited cultural heredity. Specifically, language has a set of structural *design features* such as duality of patterning (Hockett, 1960), compositionality (Krifka, 2001; Werning, Hinzen, & Machery, 2012), recursion (Hauser, Chomsky, & Fitch, 2002), and semantic convexity (Gärdenfors, 2004) that allow us to generalise from instances of language use that we have observed to novel instances. For example, it is the fact that language exhibits duality of patterning that means we can construct a large meaningful vocabulary from recombination and reuse of a relatively small collection of meaningless elements. Similarly, we can recombine and reuse these words in novel sequences to construct phrases whose meanings are composed of the meanings of their parts.

An overarching characterisation of these features is that they lead to *systematicity* in the structure of language. In simple terms, we can say that a set of behaviours exhibits systematic structure to the extent that they are interdependent rather than independent of each other. This means that a systematic set of behaviours will be describable in a way that is more concise than simply listing those behaviours. So, to take the combinatorial structure of language as an example, different lexical entries will share phonemes, and this means that the most concise description of a lexicon can be more concise than if every single word was a distinct, unanalysed sequence of unique sounds. In contrast, it is a reasonable approximation of most animal alarm-calling behaviour to say that each communicative signal in an animal’s repertoire is *independent* of the others. There is therefore no systematicity in, for example, the alarm calls of the vervet monkey (Seyfarth, Cheney, & Marler, 1980). The most concise description of the alarm-call system is simply a list of all the alarm calls.

I have linked systematicity with concise descriptions, and we will return to this idea throughout this article. For the time being it is worth saying that these descriptions can be reasonably termed *grammar* in the broadest sense of that word. The unique property of the structure of language that evolutionary linguistics tries to explain can be described as grammar, systematicity, or interdependence of behaviours, with the understanding that these terms are all essentially interchangeable. Language is shot through with this property at all levels of description, whereas it is very hard to find it elsewhere in the vast array of communicative behaviours in the rest of nature.

So, languages are uniquely systematic forms of communication, and it is this systematicity that unites the design features of language that make it an open-ended communication system (duality of patterning, compositionality, recursion, and so on). In turn, this enables our species’ greatest trick: unlimited cultural heredity. Given this, a natural assumption might be that the structure of language should be explained as the result of a process of natural selection; that the specific design features of human language arose due to selection for better communicators in the evolution of our species (Pinker & Bloom, 1990). The main message of this article is that there are problems with this type of explanation for the origins of linguistic structure. These stem from the fact that language is not only the carrier of cultural information but it is also itself transmitted culturally. I will argue that cultural transmission radically alters the evolutionary dynamics involved in the origins of language. Viewing language as a product of the interactions between individual learning, cultural transmission, and biological evolution leads us to a more nuanced picture of the origins of systematicity in behaviour. We will see that systematicity is not solely the result of natural selection for communication but rather the inevitable product of cultural transmission once certain biological prerequisites are in place.

The argument I will make is based on a series of computational models and laboratory experiments designed by myself and colleagues over the past 20 years. These are models of the cultural evolution of the design features of human language, and the interactions between cultural and biological evolution. In the next section, I survey our early attempts to model the cultural evolution of *compositionality* as arising from a process that became known as *iterated learning*. In Section 3, I turn to a considerably more general Bayesian iterated learning model of cultural evolution developed by Tom Griffiths and colleagues that allows us to test very precisely the consequences for the cultural evolution of linguistic structure of different biologically given innate constraints. In Section 4, I turn this around and ask what the consequences are for biological evolution of the cultural evolution of language. A coevolutionary model leads us to some surprising, yet robust, predictions of the nature of the language faculty. In Section 5 I look at how a number of experimental methods have been combined to give us a way of exploring cultural evolution of language in the lab that was inspired by the earlier simulation work. This has led us to a general theory of the origins of systematicity in behaviour as a trade-off between pressures from learning and pressures from use. In Section 6 I will survey experiments that aim to build bridges to the real-world implications of this theory, connecting the emergence of language in the lab to work on emerging sign languages. In Section 7 I ask how general the results are, examining an extension to a nonlinguistic task. Section 8 builds on this to look at iterated learning in other species. As a result of these comparative studies, I set out what the biological prerequisites for the cultural evolution of language might be, and suggest that *self-domestication* is the best candidate mechanism for delivering up those prerequisites in humans.

## Iterated learning models and the emergence of compositionality

It is reasonably likely that you have never before encountered the noun phrase *cyan pentagon*. Indeed, this particular pair of words does not occur even once in the entire corpus of books that Google indexes (Michel et al., 2011). I am also almost certain I have never written or said this pair of words before doing so just now. Yet I am nevertheless confident that you and I now share a common mental image evoked by this adjective–noun combination. This fact appears mundane, but would not be possible were it not for the rich *compositionality* of language. Compositionality refers to the fact that “the meaning of a complex expression is a function of the meanings of its . . . parts and the way in which they are combined” (Krifka, 2001, p. 152). Because you know what *cyan* means and what *pentagon* means and have some expectations about combinations of adjectives and nouns, you are able to interpret expressions such as the one above. This is not to say that language is completely compositional—for example, *black spot* has several interpretations that are not composed of the meanings of its parts—but nevertheless it is compositionality that gives language its open-ended expressivity, and as such is an important target for explanation in the evolutionary approach to language (Smith & Kirby, 2012).

Because compositionality is a such a fundamental and ubiquitous design feature of language, appears to be unique or near-unique to humans, and appears to be designed for the purpose of conveying novel meanings reliably, it seems plausible that this would be a target for explanation in terms of natural selection (Pinker & Bloom, 1990). In this view, a mutation or mutations occurred during our evolution that led to some individuals with the property of compositionality being specified in their faculty of language. These individuals would have a fitness advantage through being able to convey an open-ended range of meanings, ensuring that the trait would increase in frequency in the population.

**Fig. 1:**
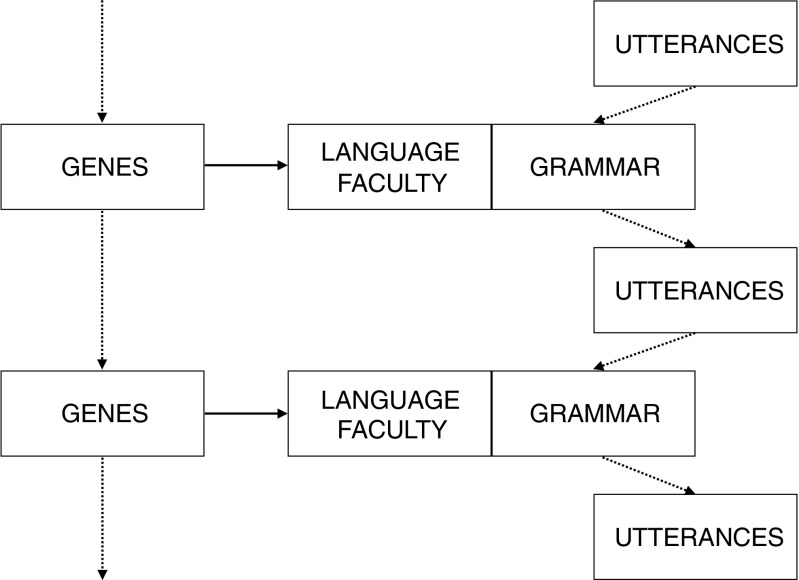
Language evolution involves dual inheritance mechanisms. The language faculty is shaped by genes that are inherited and evolve biologically. The language we speak is the product of a second, cultural inheritance mechanism as grammars arise from the interaction between our language faculty and utterances we are exposed to

A potential problem with these types of explanations is that they assume relatively straightforward connections between genetic variation, phenotypic variation, and variation in fitness. The relationship between genes and language is arguably as, or more complex than, any phenotype in nature. This is because properties of language, such as compositionality, emerge from a dual inheritance mechanism (Henrich & McElreath, 2007) involving not only genetic, but also cultural transmission (see Fig. 1). I produce sentences the way I do because my faculty of language came into contact with instances of linguistic behaviour produced by other individuals in my speech community. Whether your theory of the acquisition of language emphasises the role of linguistic data as *triggers* for parametric alternatives in the state of the language faculty (Gibson & Wexler, 1994), or treats linguistic data as input to a more general inferential learning process (Perfors, Tenenbaum, & Regier, 2011), it is clearly the case that the nature of the language we speak is the result of some combination of what we individually bring to the task of language acquisition and the nature of the data we are exposed to. Equally, the data we are exposed to is the product of other individuals who acquired their language in the same way.

Language therefore persists through a particular form of cultural transmission which we call *iterated learning* (Kirby, 2001). A behaviour is transmitted by iterated learning if it is acquired through observation of that behaviour in another individual who themselves acquired it in the same way. This much is obvious and uncontroversial. After all, we can observe this process at work during language change (Andersen, 1973; Keller, 1994). However, it is also possible that this process is involved not only in change but also in the *origins* of linguistic structure. Before we can be confident of a straightforward explanation for compositionality in terms of natural selection, we need to understand the role of iterated learning in shaping the form languages take. Before placing the explanatory burden entirely on the genetic side of the dual inheritance process, we need to know whether the cultural side could do the job just as well.

In the late 1990s, a number of researchers attempted to explain the origins of compositionality in a series of computer simulations in which cultural, rather than biological, evolution was the only mechanism (Kirby, Griffiths, & Smith, 2014; Smith, 2014). These models varied quite widely, employing very different types of learning, from connectionist approaches (Batali, 1998; Brace, Bullock, & Noble, 2015; Kirby & Hurford, 2002; Smith, Brighton, & Kirby, 2003), to models of exemplar learning (Batali, 2002), to highly symbolic approaches to grammar induction (Brighton, 2002; Brighton, Smith, & Kirby, 2005; Kirby, 2000, 2002). Nevertheless they shared the same basic structure in implementing an iterated learning process. Simulated agents learn to associate signals (usually strings of characters from an alphabet) to meanings (e.g. numeric vectors, or structured representations of some external environment) by observing a sample of the signalling behaviour of other agents in the simulation. They then go on to produce signalling behaviour themselves, which will become the training data for other agents, and so on.

**Fig. 2:**
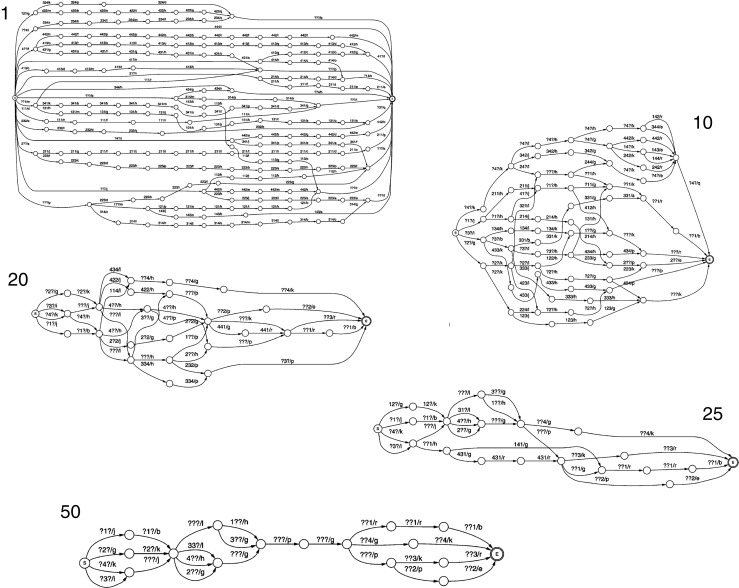
Example results from Brighton’s (2002) simulation of iterated learning. Each diagram shows a finite state transducer that maps between sequences of characters (representing signals) and feature vectors (representing meanings). Each transducer is labelled with a number indicating which generation it came from. The details are not important (and, indeed, are too small to be visible in this figure), but it is clear that transducers early in the simulation are larger and more complex than later ones. Importantly, despite this the early complex languages are less expressive. The transducers do not cover the entire possible set of meanings in the simulation. However, by the end, highly compact representations have emerged. These later languages are completely general, expressing the entire set of meanings in a compositional way such that different substrings correspond to different features of the meaning

Despite their differences in models of learning, these various simulations converged on a similar set of results. If the models are initialised with a random language—in other words, if the first agents are trained with noncompositional, random pairings of signals and meanings—then compositionality nevertheless emerges in the simulations over time as the language is passed from agent to agent. See Fig. 2 for example results from a model by Brighton (2002).

The rate of evolution of compositionality in these models turns out to be related to properties of the space of possible meanings in the simulation (Smith, Kirby, & Brighton, 2003), and what proportion of the language is observed in the lifetime of an agent (Hurford, 2002). Intuitively, this latter parameter can be thought of as the *bottleneck* on cultural transmission of language. It turns out that up to some limit, the less data the learners see (the tighter the bottleneck), the more rapidly compositionality will evolve. In the real world, the bottleneck is the limited data a child sees from which she must reconstruct the language of her speech community.^1^

For a language to persist unchanged from one generation to the next through iterated learning, it must pass through this bottleneck of use. In other words, the grammar of a language must be *reconstructable* from a sample of the possible instances of linguistic behaviour that language might produce. A “holistic” (Wray, 1998) language made up of random, idiosyncratic pairings of meanings and signals can only be learned if each and every such pairing is seen by every learner. The bottleneck needs to be wide for a holistic language to get through. On the other hand, a language in which some regularity exists in the mapping between meanings and signals—such as occurs in a compositional language—may persist even if not every meaning is observed. This is because the learner may be able to generalise from observed meaning–signal pairs to unobserved ones. Random fluctuations in the language of the population in these simulations leads eventually to regularities being observed by learners who are exposed to a subsample of the data. As learners generalise these regularities to unseen items, then the next generation of learners has clearer evidence for the existence of these regularities than the previous generation did. In this way, the bottleneck on cultural transmission leads inevitably to a cumulative increase in the generalisability of the language being transmitted (Hurford, 2000). We can therefore think of cultural transmission by iterated learning as an adaptive system, shaping language to be increasingly transmissible. Indeed, a typical analysis of iterated learning models looks at the learnability of the language at each cultural generation, measured by how similar one generation’s language is compared to the previous. The trend in these models is for language to evolve to be increasingly learnable over time.

Note that this is a subtly different perspective than the one usually found in discussions of learnability in cognitive science. Instead of asking what the *learner* needs to successfully acquire the target grammar, we ask what the *language* needs to be successfully acquired. In the former perspective, poverty of the input stimulus suggests a learnability problem. In the latter, poverty of stimulus is the *cause* of the linguistic structure that ameliorates that problem (Zuidema, 2003). Our language has a way of generalising to novel meanings such as cyan pentagons precisely because generations of language users had to try and acquire their language without seeing all possible meanings expressed. Of course, this doesn’t mean that learnability problems are solved by taking an iterated learning perspective. It is still valuable to try and understand precisely what the requirements are that the learning problem for language places on the language faculty. However, what the iterated learning perspective suggests is that we should assume that data the learner is given is optimally adapted to being learnable by whatever machinery is available to the learner. In other words, the learner can expect to be born into the best of all possible worlds for learning (Chater & Christiansen, 2010). This stands in stark contrast to some foundational results in the learnability literature (Gold, 1967), which consider the case where any grammar from a class might be the target for the learner, the worst of all possible worlds for learning.

## From biology to culture: The Bayesian approach

The results from the early models of iterated learning reviewed suggest that the best explanation for the origins of compositionality should place significant explanatory burden on cultural, rather than biological, evolution. Compositional structure emerges in a remarkably wide range of models with quite different assumptions about the nature of the learner. What they share are not specific properties of the learning model but rather the fact that behaviour is transmitted by iterated learning through a cultural bottleneck.

But there is a problem with this approach. The claim is that it is cultural rather than biological evolution that explains the origins of compositionality. A sceptical response is simply that the compositionality that emerges in the simulations is simply built into the properties of the learning algorithms in some straightforward way. If this is true, then perhaps in the real world cultural evolution is simply the mechanism whereby design features built into the biologically given language faculty are realised in the distribution of languages we see in the world. In other words, cultural evolution does no more than map transparently from properties of the biology of an individual to properties of language.

We already have some reason to doubt that this sceptical position. We know from the simulation models that the size of the bottleneck makes a difference to the process of cultural evolution, as do features of the space of meanings (Smith et al., 2003). This tells us that iterated learning does something over and above simply expressing inherent properties of the learner. The variety of different learning models, from connectionist to symbolic, that converge on the same results is also striking. Nevertheless, it is far from clear what precisely is built into many of the models of learning used in these simulations. What *exactly* is entailed about innateness from the use of a simple recurrent network in one model (Batali, 1998), and how does it relate to the built-in assumptions of a heuristically driven grammar induction in another (Kirby, 2001)? More importantly, can we make a more general claim about the relationship between innate constraints and emergent linguistic structure implied by iterated learning, or are we limited to simply running many simulations with as many different architectures as possible?

These questions led eventually to an alternative approach to modelling iterated learning, first set out in detail by Griffiths and Kalish (2007), that makes absolutely explicit the contribution of the learner to the process of cultural evolution. In line with a great deal of work in cognitive science (Chater, Tenenbaum, & Yuille, 2006), they model learning as hypothesis selection in a Bayesian framework. Learners in this approach combine *experience* (i.e. the utterances that the learner observes) with *prior inductive bias* (i.e. whatever constraints the learner brings to the task by virtue of their biology) to calculate the probability of each hypothesis (i.e. grammar). Bayes’ rule tells us how to relate these quantities:

$$ P\left(h|d\right)\propto P\left(d|h\right)P(h), $$

where *h* is the hypothesis, *d* is the set of data the learner has been exposed to, *P*(*d*|*h*) is the *likelihood* of that data given that hypothesis, and *P*(*h*) is the a priori probability of the hypothesis independently of the data seen. It is this last term, the prior bias, that corresponds to a model of what the learner’s biology brings to the task of learning. A rational learner will pick a hypothesis based on the a posteriori probability of that hypothesis given the data they have been exposed to, *P*(*h*|*d*). In this way, the Bayesian approach allows us to provide an explicit model of the contribution of the learner’s cognitive biases and predict which languages that learner is likely to acquire given some set of data.

This completely explicit approach to modelling learning bias is exactly what we want in order to escape from the problem of having a wide range of approaches to modelling learning without any clarity about what each of these approaches implies. We can simply plug in different distributions for the prior bias, *P*(*h*), and use this to change the preferences of our learners. The next step that Griffiths and Kalish (2007) took was to work out what would happen to languages passed down chains of such learners through cultural transmission. The key question here is how will having particular biases affect the outcomes of culture?

In the simplest case, we have a single agent who generates data according to their hypothesis. In other words, they will produce a set of data, *d*, that reflects the likelihood of that data given the hypothesis, *P*(*d*|*h*). A second learner, representing the next generation in an iterated learning chain, observes the data of the first learner and calculates *P*(*h*|*d*) for each hypothesis using Bayes’ rule, and uses this to pick a hypothesis, *h’*. The way this is done varies between models, in ways that have interesting consequences (Kirby, Dowman, & Griffiths, 2007). To simplify, for the moment we will assume that learners pick the best hypothesis. This is often referred to as MAP learning, because the learner is picking the hypothesis with the maximum a posteriori probability given the data. Once *h’* is selected, this second generation learner generates a set of data, *d’* (consistent with *h’*), which a third learner observes, and so on down the generations.

The key questions for this model are: What happens to *h* over time? And how does this relate to the prior, *P*(*h*)? Griffiths and Kalish (2007) suggest an approach to understanding this process in very general terms. Consider a single step of learning: The hypothesis at one generation results in a probability distribution over hypotheses at the next generation. This gives us a *transition matrix,* giving the probability of iterated learning taking us from any one hypothesis to any other.^2^ Various parameters potentially shape the values in this transition matrix—most notably the prior bias, *P*(*h*), and the bottleneck (i.e. how much data the learner is exposed to).

Once we have a transition matrix, we want to derive the consequences of iterated learning for the distribution of languages. In particular, we are interested in the *stationary distribution* of languages. This is the probability distribution over hypotheses that we expect to see once cultural evolution has run for enough generations for the influence of the initial hypothesis of the first learner to no longer be felt. Assuming that language has been around on the planet for long enough that there is no longer any trace of the first language spoken, we can equate *stationary distribution* and *language universals.* 3

We can mathematically derive the stationary distribution from a transition matrix with some basic linear algebra, and this therefore allows us to make general predictions about the relationship between the prior bias of the learner, *P*(*h*), and the actual distribution of cross-linguistic variation this would give rise to. In other words, Bayesian iterated learning gives us a set of modelling tools which allow us to precisely understand what cultural evolution contributes to the explanation of language universals over and above what is built into our model of learning.

Kirby, Dowman, and Griffiths (2007) work through a simple example of Bayesian iterated learning to understand the relationship between what is built into the learner and what is the outcome of cultural evolution. They use morphological regularity as their example. Consider a space of meanings that a language needs to convey. Meanings can be expressed either using a regular paradigm or as irregular exceptions. What is the relationship between whatever prior bias the learners have in favour of regularity and the distribution of regularity we see in the stationary distribution?

**Fig. 3:**
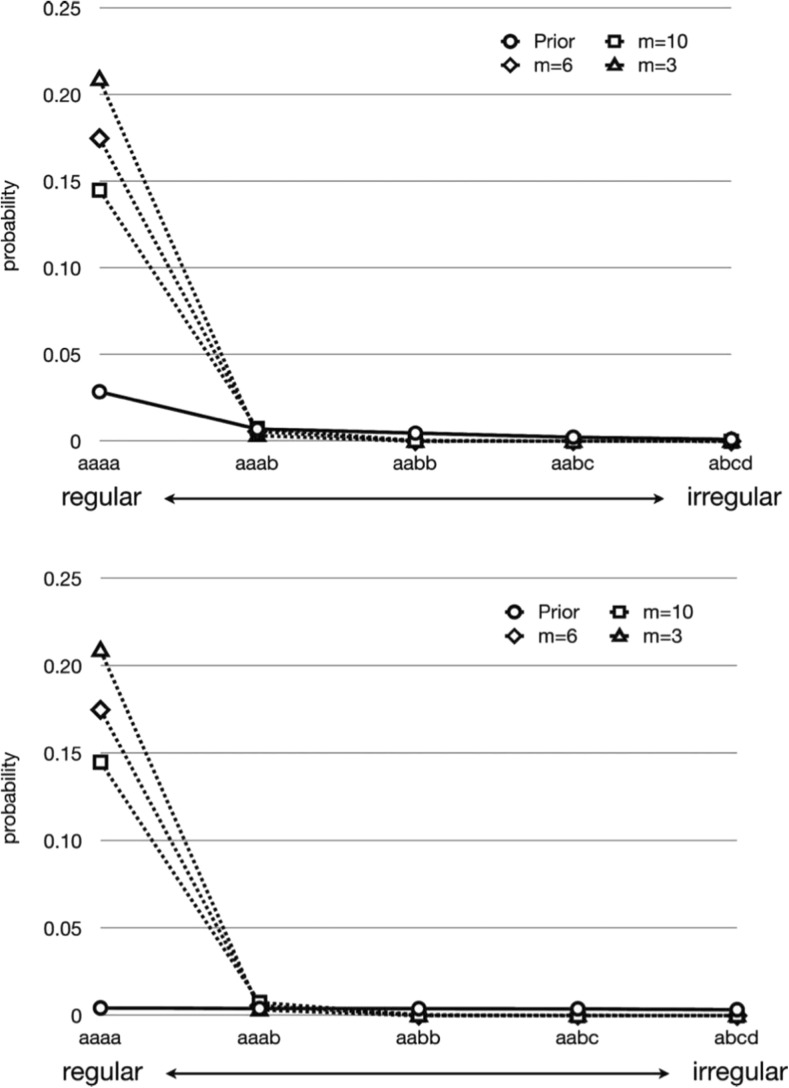
Results from the Bayesian iterated learning model in Kirby et al. (2007). Languages vary in their regularity in this model, and five examples are shown in order of decreasing regularity. The solid line represents the prior bias of the learners favouring regularity. In the top graph, the bias is relatively strong, whereas in the bottom it is vanishingly small (all languages have almost but not quite the same prior probability). The various dashed lines indicate the distribution of languages that arise from cultural evolution with different amounts of training data each generation. As the number of training examples (*m*) decreases, the bottleneck on transmission tightens and the prior preference for regular languages is amplified. However, different prior bias strengths have no effect on the distribution of languages that emerges

Figure 3 shows results of this model for different strengths of prior bias, and different amounts of training data (i.e. the bottleneck on cultural transmission). These results show very clearly that while the *existence* of a bias in favour of regularity is important (otherwise there would be nothing in the model distinguishing the different types of languages), the *strength* of this bias makes no difference. In addition, languages in the stationary distribution appear far more regular than the prior bias would suggest. Cultural evolution amplifies the biases of the learner. What does determine how regular the languages become is the width of the bottleneck. The less data the learners see, the more regularity is favoured.

This result is one from a range of studies of Bayesian iterated learning, varying various parameters of the models including the hypothesis selection strategy (Griffiths & Kalish, 2007), the size of the population of learners (Burkett & Griffiths, 2010; K. Smith, 2009), and structure in the space of meanings (Perfors & Navarro, 2014). In most, but not all (Kalish, Griffiths, & Lewandowsky, 2007), models the stationary distribution is influenced by the prior bias of the learners but does not mirror it perfectly.

The implications of these results for how we go about explaining the origins of linguistic structure are far reaching. They demonstrate that we cannot be sure that any particular theory of the language faculty will make the right predictions about the nature of language without embedding that theory in a model of culture. Only by doing so will we be able to figure out the long-run consequences the language faculty has for the dynamics of cultural evolution. For example, faced with a completely universal property of language, such as compositionality, we might be tempted to build that into our model of the language faculty as a hard constraint, leading us to seek an explanation in terms of the biological evolution of the language faculty for such a constraint on the nature of language. However, it turns out that such a hard constraint is not required. It may be that only soft (and domain general) biases in favour of simplicity are required (Culbertson & Kirby, 2015) given that iterated learning in the presence of a bottleneck is sufficient to amplify these biases. Similarly, we cannot “read off” properties of the language faculty straightforwardly from our observations of the nature of language.

The lesson is that we should be careful not to seek simple mappings between language universals and the language faculty. Languages adapt culturally as an inevitable consequence of iterated learning in such a way that over time they become optimised for transmissibility. The tougher the transmission bottleneck, the more pressure there is on language to adapt.

However, it is also important to emphasise that the results of Bayesian iterated learning do not imply that biology has no role to play in the explanation of language structure. On the contrary, this work places the prior bias right at the centre of the picture. In this view, individual cognition and cultural transmission interact. Linguistic structure is the emergent consequence of that interaction.

## From culture to biology: Evolutionary implications of iterated learning

In Section 2, I pointed out that language evolution can be seen as arising from a dual inheritance process. Both cultural and biological evolution are potentially involved in shaping language. Nevertheless, up to this point we have said nothing about biological evolution. The Bayesian iterated learning model enables us to characterise precisely how the nature of language emerges from a population with particular cognitive biases through cultural evolution, but remains silent about how those cognitive biases got there in the first place, or what types of bias we should expect. In this section, I will briefly survey how the Bayesian iterated learning model can be expanded to include biological evolution, effectively giving us a complete picture of how individual learning, cultural transmission, and biological evolution interact.

We want to understand what kind of language faculty is likely to evolve, given what we now know about the process of cultural evolution through iterated learning. In Bayesian terms we can ask how the prior bias will evolve under selection. We treat the prior bias as something determined by our genes, and accordingly create populations of language learners who inherit their prior bias from their parents. We implement iterated learning in the standard way by allowing individuals to learn their language from others in the population. Finally, the fitness of a learner is determined by how well they have learned the language of the population. This can be seen as a proxy for how well learners will communicate within their speech community.^4^

Thompson, Kirby, and Smith (2016) set out a model of this coevolutionary process and examine its behaviour under a wide range of assumptions. Here, I will describe the simplest form of their model. The model consists of a series of generations, with multiple agents at each generation. Each agent learns a language from data produced by the previous generation (in the simplest case, each agent receives data from a randomly chosen single agent in the previous generation). Fitness is assigned by how closely an agent’s language after learning is complete matches the languages of the other agents in the same generation. The fittest individuals pass on their genes to the next generation, with some small chance of mutation in the genes.

In the simplest form of this simulation, there are just two types of language (language Type 0, and language Type 1). Each type of language gives rise to utterances that unambiguously indicate the language being spoken, except that there is a small chance of noise, meaning that the speaker may produce an utterance indicative of the wrong language type. The prior bias, and hence the model of the innate language faculty, in this model can be expressed as a single number between 0 and 1, giving the prior probability assigned to languages of Type 1. So, a prior bias greater than 0.5 indicates a learner who prefers languages of Type 1, whereas a prior less than 0.5 indicates a preference for Type 0. The closer to 0 or 1 that the prior is, the closer the model is to implementing a hard, inviolable constraint.

In the model, this continuous bias arises from the additive effect of multiple discrete genes, each contributing a small amount to the bias. A set of genes all indicating a preference for languages of Type 1 will lead to a prior bias of 1, whereas a mix of Type-1 favouring and Type-0 favouring genes will lead to a prior bias of 0.5. This scheme means that any bias is possible (within some granularity defined by the total number of genes) but maintaining a strong bias against mutation pressure requires selection for that bias. To put it another way, random drift will tend to lead to a mix of genes favouring Type 1 and genes favouring Type 0, pulling the prior towards 0.5.

**Fig. 4:**
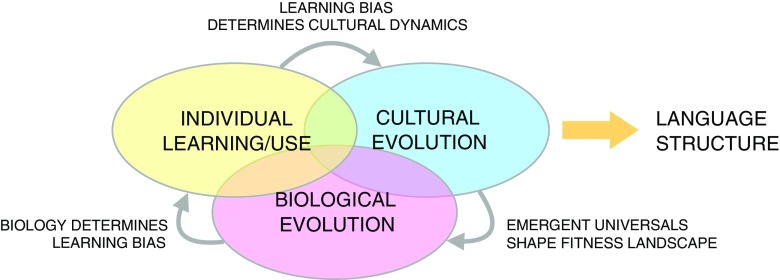
Language structure arises from a complex set of interactions between three complex adaptive systems operating at different scales

With all these elements in place, we have a very simple model that incorporates individual learning of languages, cultural evolution of languages, and biological evolution of the learners. Notice that there are interactions between all three of these. The way agents learn is shaped by their genes. The distribution of languages that emerge is determined in part by the innate biases of the learners. Finally, the fitness of the learners is dependent on the languages spoken in the population. This interlocking set of complex adaptive systems operating at different timescales is what gives rise to language (see Fig. 4).

**Fig. 5:**
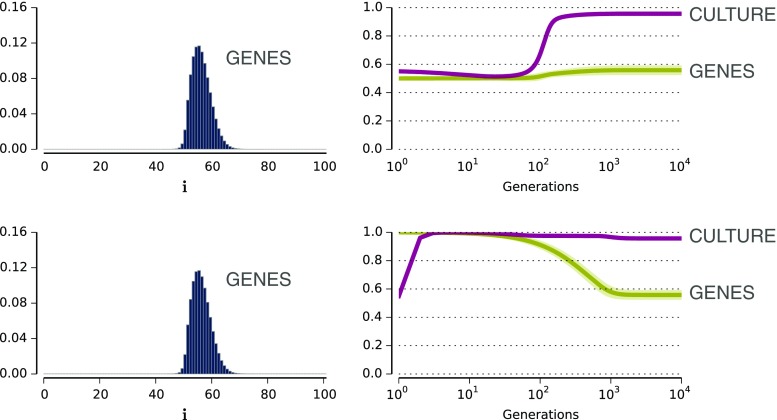
Results from the coevolutionary model with two different starting points (Thompson et al., 2016). The plots on the left show the distribution of genes that evolve, with high or low values of “i” on the *x*-axis indicating a strong constraint in favour of Type 1 or Type 0 languages, respectively, and a value of 50 indicating no bias. The plots on the right show time courses indicating how the genes of the population evolve (values greater than 0.5 indicating a bias in favour of Type 1), and how the languages evolve culturally (high values indicating a prevalence of languages of Type 1 in the population). The top row shows the outcome of gene-culture coevolution with an initial population of unbiased learners and a very slight majority of languages of Type 1 being spoken in the population. The bottom row shows the outcome with an initial population of strongly constrained learners. In both cases the outcome is the same: a strong universal preference for languages of Type 1 in the culture, but the weakest possible bias in favour of languages of Type 1 in the language faculty of the learners. (Thanks to Bill Thompson for the graph; for more details and explanation, see Thompson, 2015)

Figure 5 shows the results of running a numerical version of the model^5^ with two starting conditions: a strongly constraining population, in which every agent is born only being able to acquire languages of Type 1, and a “blank slate” population, in which learners have no a priori preference for either type of language. In both cases, the initial culture, that is, the data that the very first generation learns from, is one in which both types of language are spoken in nearly equal proportion, with a small preference for Type 1. Despite their different starting conditions, the end result of these simulations is identical: a population with a very weak bias in favour of languages of Type 1, but one in which almost everyone speaks languages of Type 1.

The combination of learning, culture, and evolution leads to predictions about language universals and the language faculty that are not immediately obvious. We predict the emergence of strong universals in the population—languages will appear to vary within a set of hard constraints—but these universals will be supported by the *weakest possible innate predisposition*. Thompson et al., (2016) test a very wide range of models with different assumptions about the nature of language, population structure, fitness pressures, and so on, leading them to the conclusion that this is a very general result, robust to variation in parameters, and not limited to particular features of their particular model.

This result appears to be the inevitable consequence of cultural transmission intervening between genotype and ultimate phenotype for language. We can run the same model again, but with culture turned off, so that the language that a learner acquires is determined entirely by their prior biases (but fitness is still calculated in the same way and will be dependent on the frequency of different language genes in the population). Only in this acultural scenario do strong innate constraints evolve.

The take-home message of these results, and related models of the interactions between learning and evolution (Chater, Reali, & Christiansen, 2009; Kirby & Hurford, 1997; Smith & Kirby, 2008), is that *culture matters*. These models suggest that the addition of cultural evolution profoundly alters the way in which biological evolution proceeds. It provides an example of *masking*, an often neglected force in evolution that Deacon (2010) argues is particularly important in human evolution, in which the relationship between genetic variation and its effects on fitness can change. Iterated learning amplifies small biases, leading them to have large effects on the distribution of languages that are the result of cultural evolution. Because of this, small deviations from neutrality arising from genetic variation are highly visible to natural selection due to their large ultimate phenotypic effects. In other words, culture *unmasks* small variations in the language faculty, potentially making innate biases more evolvable.

Crucially, however, there is a flip side to this unmasking effect. The amplification of biases that are the result of cultural evolution simultaneously *mask* the strength of biases from the view of natural selection. Variation in the genes encoding strength of bias simply have no fitness consequences. Cultural evolution leads to the same phenotypic outcome, the same distribution of languages, whether the agents have a weak, defeasible preference or a hard, inviolable constraint. The inevitable result is a degradation of strong constraints due to masking.

These results suggest, on purely evolutionary grounds, that we should expect the language faculty—or at least the part of the language faculty involved in language acquisition—to take the form of weak biases to the extent that those biases have evolved under selection for communication. This should be our default assumption, even in cases where we see evidence for strong constraints at the level of actual linguistic variation. To turn this argument on its head, if we have good independent evidence that there *are* some hard constraints on the acquisition of language, it is unlikely that those constraints are the result of natural selection for communication.

Put simply, taking culture seriously in an evolutionary model has lead us to the conclusion that the language faculty may contain domain-specific constraints only if they are weak, and strong constraints only if they are domain general.

## Language evolution in the lab

The models I have covered so far suggest that a culturally transmitted system will spontaneously adapt to aid its own survival through iterated learning. Changes introduced into the evolving language at each generation will persist to the extent that they are learnable by subsequent generations. The inevitable result appears to be a cumulative increase in learnability of the transmitted system.

Wherever we see results in abstract simulations, a reasonable concern is whether they also apply straightforwardly in the real world. In the case of these models of language evolution, we can wonder whether human biases are similar to the ones we build into our models, and whether it is reasonable to imagine that languages adapting to increase their learnability by becoming more structured is something that would happen on a sensible timescale outside of computer simulation.

These concerns have lead over the past decade or so to a number of attempts to recreate the iterated learning process in the psychology experiment lab with real human agents. The idea is essentially to combine artificial language learning experiments from psycholinguistics (Reber, 1967) with diffusion chain and artificial microsociety paradigms from experimental cultural evolution (Mesoudi & Whiten, 2008). We can then ask whether we can observe the spontaneous creation of linguistic structure through cultural transmission and whether this mirrors the predictions of our models.

The general design of these studies can be exemplified by a simple drawing experiment by Tamariz and Kirby (2015). In this study, a modification of the classic serial reproduction task of Bartlett (1932), participants were shown an abstract drawing on a sheet of paper and then asked to recreate it on another sheet of paper. Their drawing was then shown to the next participant in the experiment, who was asked to recreate the drawing on a third sheet, and so on. Tamariz and Kirby (2015) ran two conditions of the experiment—the copy condition, in which the drawing being recreated remained in view while it was copied, and the memory condition, in which the picture was removed from view as soon as participants started drawing. In both conditions, errors in the reproduction process led to the drawings evolving gradually over the “generations” in the experiment. Different chains in the experiment diverged from the same starting drawing in a way analogous to the distinct lineages that evolve in a wide variety of cultural domains in the real world. However, the structure of the drawings differed consistently in the two conditions. Specifically, the drawings in the memory condition evolved cumulatively to be *simpler* than those in the copy condition, whose complexity remained at the same level as the original. Tamariz and Kirby (2015) quantify this by measuring the compressibility of the bitmap files created by scanning the drawings. Remarkably, the file size of the drawings created by a variety of compression algorithms decreased consistently over generations in the memory condition, but not in the copy condition.

This result demonstrates that cultural evolution does not merely lead to drift through copying error. The memory condition, by making participants map the external drawing into some internal representation and then out again, enforces a bottleneck on the cultural transmission process that does not exist in the copy condition. The inevitable result is that the drawings adapt to this bottleneck. Simpler, more compressible drawings are easier for participants to hold in memory than complex, incompressible ones, and this acts as a bias that applies at some point in the process between seeing the original drawing and reproducing it. This bias may be so small that it is undetectable at the level of one instance of copying, but over time it is amplified by the iterated copying process, leading to the results that we see at the end of the chains.

What Tamariz and Kirby (2015) do for drawings can also be applied to miniature artificial languages, effectively extending the methods of artificial language learning from learning in a single generation to the cultural transmission of language over multiple generations. The question is whether the kinds of systematic structure that we are interested in (e.g. compositionality) can be shown to emerge in the lab in a way similar to what is seen in the models reviewed in the previous sections.

In the first of these experiments, Kirby, Cornish, and Smith (2008) had participants learn a miniature artificial language in which strings of syllables labelled coloured moving shapes. In all there were three colours, three shapes, and three movements, leading to 27 possible scenes to be labelled. The initial language consisted of entirely random strings so there was no systematicity in the relationship between signals and meanings. After being exposed to half of this language, participants were asked to label the full set of 27 scenes (i.e. they had to recall the strings they were trained on and also generalise to unseen meanings). The resulting language from the first participant became the language that was used to train the second participant in the experiment (with a different selection of items withheld from training). This process was repeated for 10 “generations” in four separate chains from different random initial languages.

Following the previous body of simulation studies, Kirby et al. (2008) predicted that the languages would evolve to be increasingly learnable over generations in the experiment, and that they would do this by virtue of becoming compositionally structured. This is expected because there is a bottleneck on the transmission of the languages imposed by withholding half of the data. However, the result of the experiment *didn’t* match what was expected from the simulations. Although the languages did indeed become more learnable over generations, they did not do so by becoming compositional. The initial random language is strictly unlearnable—there is no way a participant in the experiment could possibly correctly generalise to unseen meanings. This is generally true for the first few generations of the experiment. At this stage, the language is changing rapidly as learners introduce errors in the transmission as a result of the unlearnability of the language. In contrast, the final generations of the experiment are typified by highly learnable—and therefore stable—languages which permit accurate generalisation to unseen meanings. In this sense, they appear just like the results of the simulation models. Where they differ is the precise way in which the languages have evolved to maximise learnability.

**Fig. 6:**
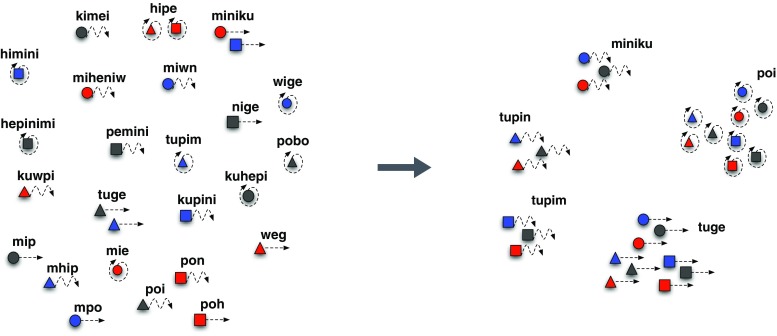
Example languages from Kirby, Cornish. and Smith’s (2008) experimental iterated learning study. The language on the left is the output of the first learner in a transmission chain, after they attempted to learn an initial randomly generated language. In this experiment, the language labelled 27 different scenes involving different coloured shapes moving. The language changed gradually as it was passed down 10 generations of learners to become the one indicated on the right. The same 27 scenes are now labelled with only five words, which carve up the space in systematic ways, for example by movement. The language on the left is unlearnable from a subsample, whereas the language on the right is easy to learn. (Colour figure online)

Rather than becoming compositionally structured, the languages became highly ambiguous (see Fig. 6). Whereas the initial random languages had 27 distinct strings—one for each meaning—the final languages had as few as two. The languages had become more learnable by simply jettisoning words. Interestingly, they did this in a nonrandom way. Words would map onto regions of the set of meanings in such a way that they systematically underspecified certain features of the scenes. For example, a language might evolve that simply did not encode the colour dimension, or which collapsed the distinction between shapes for a particular category of movements. This *systematic underspecification* is an adaptation by language to the challenge of being transmitted through a bottleneck, but it is one that comes at the cost of the *expressivity* of the language.

In retrospect, this is perhaps an obvious outcome. The most learnable language of all is after all a highly *degenerate* one in which every meaning is expressed by a single word. Given that the only process in our iterated learning experiment is learning, this is the only pressure operating on the evolving language. The languages inevitably evolve to reflect our prior bias, and in this experiment this appears to strongly favour simpler, more compressible, languages—languages that underspecify in a systematic way.

So where does this leave compositionality? I suggest that compositionality is not the result merely of a pressure for learnability, and hence simplicity, but an interaction of that simplicity pressure and a partially countervailing pressure favouring languages that are *expressive*. The obvious source of an expressivity pressure is from communication (Fay, Garrod, Roberts, & Swoboda, 2010; Galantucci, 2005; Garrod, Fay, Lee, Oberlander, & Macleod, 2007). To test this idea, Kirby et al. (2015) and Winters et al. (2015) extend the iterated learning experiments to include interaction as well as learning. Instead of a single participant at each generation, two participants learn the same language and then take turns playing a communication game over networked computer terminals. In this game, they alternately play as *director* and *matcher*. The director has to send a signal that will allow the matcher to pick out a particular scene from an array of alternatives. At the end of multiple rounds of the communication game, the last labels for each scene used by one of the participants is used as the language that is passed on to the next pair of participants in the chain.

**Fig. 7:**
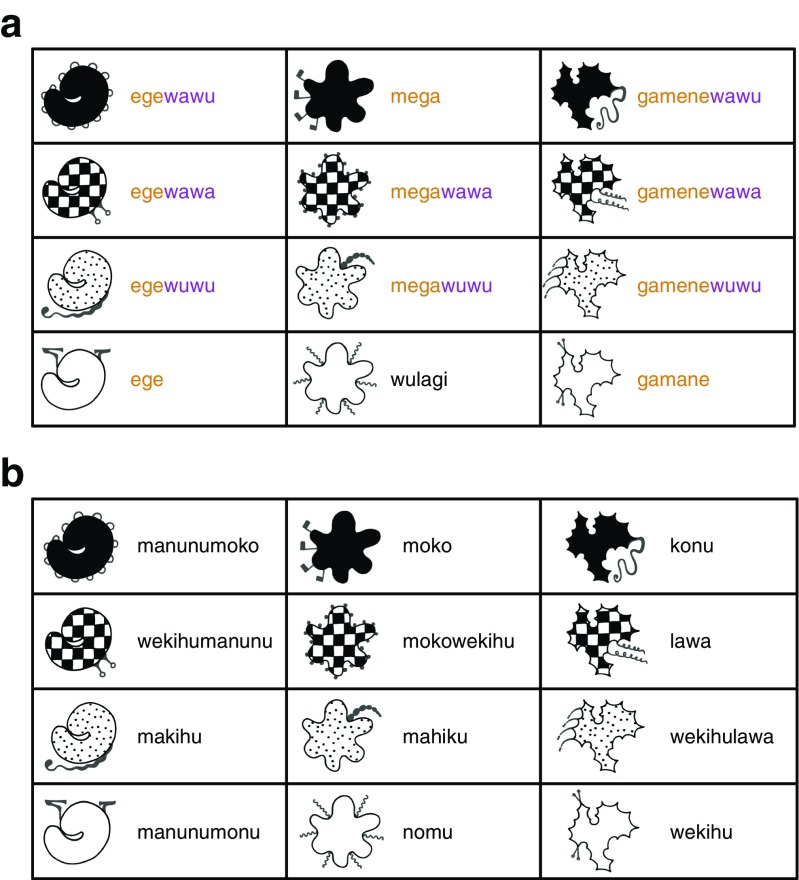
Example languages from Kirby, Tamariz,, and Smith’s (2015) experiment involving both interaction and transmission to new learners (A), and interaction alone (B). Compositional structure emerges in the former case where both learning and communication put pressure on the language to be both compressible and expressive. Here, the language uses a prefix to indicate shape and a suffix to indicate the fill texture. When only interaction is involved, a largely uncompressible, holistic language emerges

In this new version of the experiment, we have iterated learning and dyadic interaction both potentially shaping the language as it evolves over time in the experiment. The result is strikingly different from that found in the first experiment described. Rather than a *degenerate* language, a *compositional* one evolves. In Kirby et al. (2015), the scenes are three different shapes with four different textures applied to them, and languages evolve that have prefixes corresponding to shapes and suffixes corresponding to texture (or vice versa). These compositional languages are more learnable than the initial random *holistic* ones, but unlike the degenerate languages in the previous experiment, they are also expressive (see Fig. 7a).

Is this result due to the combination of iterated learning and dyadic interaction, or is dyadic interaction alone sufficient? To test this, Kirby et al. (2015) run the experiment again, but rather than replacing participants at each generation, they keep the same pair of participants in the lab throughout. In this way, we can isolate the effect of repeated transmission to learners on the evolution of the language.

The result is that the original random holistic language, which is already fully expressive because a distinct string is associated with each meaning, does not evolve much at all (see Fig. 7b). The language neither becomes compositional, nor does it degenerate. It seems that compositionality only emerges in these experiments where there is both a pressure to be simple, driven by learning, and a pressure to be expressive, driven by communication. With only the former pressure, we get highly compressible degenerate languages; with only the latter, we get noncompressible, expressive, holistic languages.

At this point it is worth addressing a justifiable concern about this kind of study. Our aim is to illuminate the forces at play in the origin of design features of human language, such as compositionality, by using human participants in a miniature language transmission experiment. But all our participants already have a language. How do we know that what we are seeing expressed in the experiments is a result of cultural transmission amplifying fundamental learning biases, or simply a reflection of *acquired* bias? To put it bluntly, are the resulting languages compositional because our participants already speak compositional languages?

**Fig. 8:**
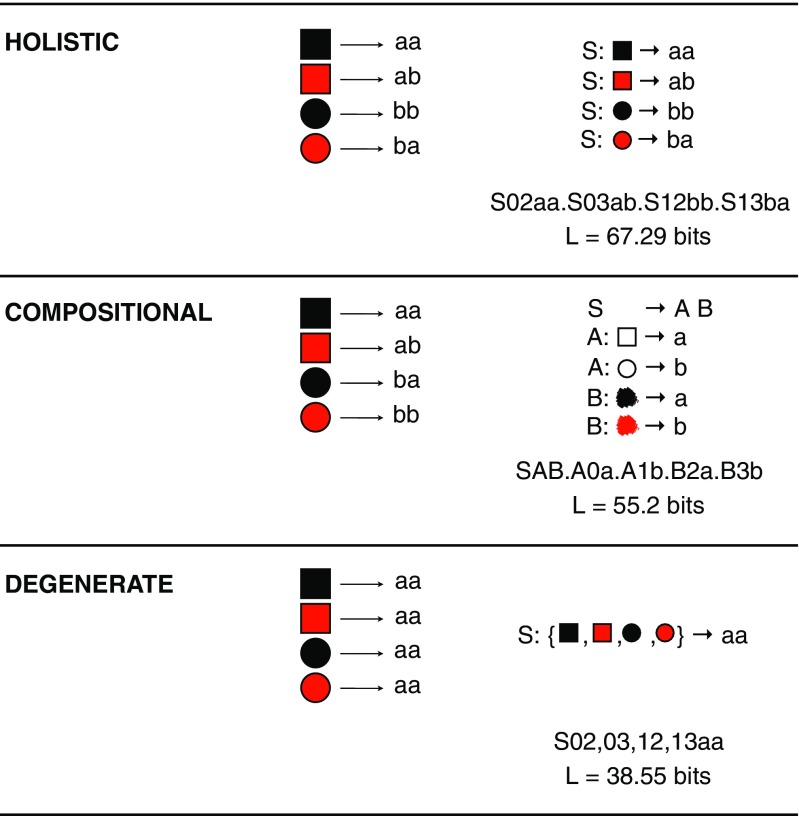
Languages from the simulation in Kirby et al. (2015). Each language maps between a simple meaning space (consisting of two binary features, here represented as colour and shape) and a signal space (consisting of strings of length, two from an alphabet of two characters). The prior for each language is based on the coding length of the language in bits (L). This is computed by first representing the language as a grammar with semantic annotations, and then encoding that grammar in a minimally redundant form as a string of characters. The coding length of that string reflects our intuitions that a degenerate language with a single word is simpler than a holistic language with a distinct word for each meaning, and that the complexity of a compositional language lies between these two extremes

To address this, Kirby et al. (2015) replicate their experimental results in a Bayesian iterated learning model in which there are generations of pairs of agents that interact and learn from each other, just as in the experiments. Of course, the advantage of a simulation over an experiment is that we can be explicit about the source of bias. The agents are exposed to signal-meaning pairs and acquire a language that is defined as a mapping from meanings to signals. Crucially, Kirby et al. (2015) implement a very general simplicity bias (Chater & Vitányi, 2003), based on an approximation of the Kolmogorov complexity of the languages. Specifically, following Brighton (2002), agents prefer languages that can be expressed as concise context-free transducers (see Fig. 8). During interaction, agents have an additional bias against signals that would not discriminate the target meaning in context (Frank & Goodman, 2012).

The results of the simulation match the experimental findings closely. Without interaction, iterated learning alone leads to the simplest possible language. In other words, degenerate languages with only a single word for all meanings. Without transmission to new learners, interaction alone leads to expressive but incompressible languages in which every meaning is labelled with a distinct string. Only when both interaction and transmission to new learners are in play in the model does compositionality evolve as a solution to the problem of jointly optimising expressivity and learnability. These results suggest that the emergence of compositionality in our experiments is not a simple reflection of the fact that our participants already speak a compositional language.

## From lab to the real world

The experiments and models described above suggest that cultural transmission of language leads inevitably to more and more compressible languages (within certain expressivity limits derived from the use of language for discrimination between meanings). The hypothesised prior bias in favour of simplicity/compressibility is extremely general (Chater & Vitányi, 2003)—arguably a feature of any reasonable learning system. Nevertheless, a domain-general bias such as this can have domain-specific effects. Culbertson and Kirby (2015) point out that the same underlying preference for simplicity can have quite varied effects in different domains of language as it interacts with diverse linguistic representations. For example, the simplicity bias can favour compositionality, as seen above, combinatoriality (Verhoef, 2012), phonological structure (Wedel, 2012), regularisation (Smith & Wonnacott, 2010), cross-category harmony (Culbertson, Smolensky, & Legendre, 2012), scope isomorphic ordering principles (Culbertson & Adger, 2014), kinship systems (Kemp & Regier, 2012), colour terms (Xu, Dowman, & Griffiths, 2013), spatial vocabulary (Carstensen, Xu, Smith, & Regier, 2015), semantic convexity (Carr, Smith, Cornish, & Kirby, 2016; Gärdenfors, 2004), and numeral systems (Xu & Regier, 2014). Linguistic properties such as these may be the inevitable result of adaptation of languages through iterated learning.

There is, however, something a little peculiar about this argument. It seems to suggest that the cultural evolution of language is one of cumulative decrease in complexity. The starting point in the compositionality experiments is provided by the experimenter and involves every meaning being conveyed by a distinct random signal. This initial random language is simply unlearnable. This is a reasonable starting point for an experiment, perhaps, but it begs the question of what this random, uncompressible, complex, holistic initial language corresponds to in the real world. To put it another way, what is a reasonable model of the very first language prior to any cultural evolution having taken place?

Answering this question might appear hopeless. If we assume that all existing languages had a common ancestor, that ancestor is lost in prehistory, and languages do not fossilise. This belief—of the impossibility of studying an ultimate progenitor language—is surprisingly widespread in the field of language evolution. Fortunately, the premise is quite clearly false: All existing languages *do not* share a common ancestor. Language has sprung forth multiple times in history. We know this because we have exceptionally well-documented cases of it having done so very recently, giving us the opportunity to observe possible initial conditions for the cultural evolution of language directly.

This evidence comes from emerging sign languages such as Nicaraguan Sign Language (Senghas, Kita, & Ozyürek, 2004), Al-Sayyid Bedouin Sign Language (Aronoff, Meir, & Sandler, 2005), Kata Kolok (de Vos, 2015), and Adamarobe Sign Language (Nyst, 2007), among others. In these cases, a sufficient number of deaf individuals without access to an existing language are brought together either through the formation of deaf schools and clubs or by an increase in genes for deafness in a closed population (Meir, Israel, Sandler, Padden, & Aronoff, 2012). The spontaneous creation of a new sign language appears to be the inevitable consequence of this kind of situation. This, therefore, provides us with extraordinarily precious instances of the evolution of language in the real world.

Now, of course, this can only tell us about the process of cultural evolution. All the individuals involved in the emergence of these new languages are obviously fully modern humans! The extent to which significant coevolution involving both genetic changes and cultural changes occurred in the origins of the very first languages is an open question, and the goal of the models of Section 4 is to begin to explore the consequences of any such coevolution. Relatedly, there is vigorous debate about whether there was a stage in the evolution of language in which an ancestor of ours possessed something that was not quite a fully modern language faculty, and as a result spoke a protolanguage that was not quite a fully modern language (Arbib & Bickerton, 2010; Jackendoff, 1999).

Nevertheless, it would be wrong to dismiss emerging sign language data as being irrelevant to the issue of language evolution. It shows us that cultural processes in a population of individuals possessing a modern language faculty can create a language where one previously did not exist, and, furthermore, it shows us precisely the steps involved in doing so.

In the Nicaraguan Sign Language case (Senghas et al., 2004), we can discern at least three stages in the creation of the language. First, there are multiple independent instances of the creation of *homesign* in a geographically dispersed population of isolated deaf children. Homesign is a method of communication that deaf children of hearing parents use if they are only exposed to cospeech gesture. Second, multiple homesigning children are brought together in a cohort in a deaf school and begin forming conventions of communicating with one another. Finally, new cohorts of individuals join the growing community, are exposed to the existing conventions being formed by the previous cohort, and transform these conventions through learning.

There are, therefore, three processes involved in the creation of a language like Nicaraguan Sign Language. There is the initial *improvisation* of homesign (Goldin-Meadow & Brentari, 2015), *interaction* between signing individuals, and *iterated learning* as the emerging language is passed down through cohorts. The experimental methods described in Section 5 allow us to examine the contribution of *interaction* and *iteration* to the emergence of systematic structure, but do not consider the role of *improvisation*, which is why we ended up with the slightly awkward step of constructing an initial random language for the first participants in a chain to learn.

Fortunately, there is a growing experimental literature on improvisation of communicative behaviour in the gestural domain (Christensen, Fusaroli, & Tylén, 2016; Goldin-Meadow, So, Ozyürek, & Mylander, 2008; Schouwstra & de Swart, 2014). The so-called silent gesture paradigm looks at how hearing adults who don’t know any sign language convey various concepts using only their hands. The focus of much of the work in this area has been on uncovering biases in the way events are conveyed, and this research has demonstrated convincingly that there is limited, if any, influence from participants’ native language on the ordering of elements in improvised gesture.

If silent gesture can be used as a stand in for the early, improvised forms that language might take (e.g. in homesign), then a reasonable approach would be to combine this method with the experimental iterated learning techniques described in Section 5. Smith, Abramova, Cartmill, and Kirby (2016) look at the way in which complex events are represented in an experimentally evolving miniature sign language. The initial input to the transmission chains in this experiment consisted of improvised gestures conveying events depicted by 16 animations of a moving ball. The ball animations were designed to each exhibit one of four possible paths (the ball could be moving in an *s* shape, in a circle, diagonally, or horizontally), and simultaneously exhibit one of four possible manners (the ball could be sliding, spinning, rolling, or jittering). Consistent with data from cospeech gesture (Senghas et al., 2004) and other silent-gesture experiments (Clay, Pople, Hood, & Kita, 2014), improvised gestures almost universally conveyed these two aspects of the event simultaneously.

The next stage of the experiment involved new participants learning to produce gestures on the basis of the previous, improvised, gestures. The recorded gestures of these participants became the target for a second generation of learners and so on in the standard fashion for an iterated learning experiment. Over generations, the gestures became more systematic and compressible. In some chains, the previously simultaneous gestures for the complex events became segmented and linearised, with manner being expressed separately, followed by path. In this sense, the languages became less iconic, but more systematic (because the exact same gesture is reused across multiple events). This finding mirrors the way manner/path separation emerges in the second and third cohorts of Nicaraguan Sign Language (Senghas et al., 2004).

## How general is the evolution of systematicity?

Throughout this article I have emphasised the idea that cultural evolution naturally leads to systematicity in language, and that this is driven by a general prior bias for simple, compressible representations of the world being amplified by transmission through iterated learning. I have shown how this may explain the origins of some fundamental design features of language, and noted that it might also lead to more specifically linguistic universals, such as word-order harmony, as the domain-general bias for simplicity interacts with linguistic representations (Culbertson & Kirby, 2015).

All of the experiments described so far have framed the task that participants are undertaking in linguistic or communicative terms. For example, the original iterated learning experiments referred to the task as one of “learning an alien language”; the interaction experiments are set up to elicit explicitly communicative behaviour from pairs of participants; and so on. This leaves open the question whether the systematicity we see emerging in these experiments requires a linguistic framing—whether the bias in favour of systematicity is specific to language.

Additionally, one could argue that the design of many of these experiments might lead participants to treat the behaviour they are being exposed to as a system right from the outset, rather than a collection of independent behaviours to be learned. In other words, the systematicity that emerges might be as much a product of how these experiments are *framed* as it is about a general tendency for cultural evolution to lead to systematic structure.

To address this concern, Cornish, Smith, and Kirby (2013) designed an iterated sequence learning experiment in which participants perform a series of independent memory tasks, each presented in the form of a familiar sequence recall game: the “Simon” game. In Cornish et al.’s (2013) version of the game, a sequence of coloured lights is presented on a touch screen interface, after which participants simply have to immediately recall the sequence they just saw. Each participant played 120 of these games, consisting of 60 sequences presented in two blocks. Participants were organised into chains such that the first participant was presented with 60 random sequences (each of length 12), and subsequent participants were presented with the sequences produced by the previous participant in the chain.

**Fig. 9:**
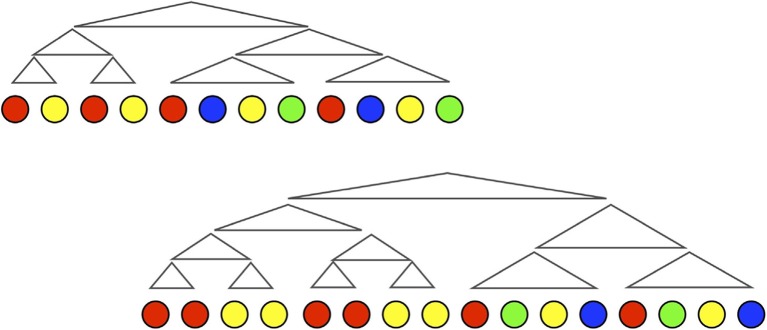
Two sequences drawn from the final set of 60 sequences in the last generation of one of the chains in Cornish et al.’s (2013) experiment. Note how the structure of the two is similar, with the exception that the second involved a doubling of the elements in the first part of the sequence. The hierarchical structure shown is suggested by the parallels between these and other sequences in the set. (Colour figure online)

In this way, 60 lineages of sequences were created for each chain of participants run in the experiment. Just like in other iterated learning experiments, participants in later generations in a chain found the task easier than earlier participants. This was a result of the sequences evolving to be easier to remember as collections of chunks (see Fig. 9). Note that this experiment was framed as a series of independent imitation games, with no inherent expectation of the 60 games being systematically related to each other. Nevertheless, systematic interdependence of sequences emerges in this experiment. The set of sequences is more compressible in later generations, and a new set of participants tested on the sequences find them harder to imitate if they are drawn from different chains than if they are drawn from the same chain.

As in previous iterated learning experiments, the set of behaviours being transmitted adapts to maximise its own accuracy of transmission. In this case, sequences that share features in common are easier to copy than sequences that are all distinct since chunks that are learned from one sequence are easier to recall if they also appear in a later one.^6^

The fact that global systematicity can emerge even in an experiment design that involves immediate recall rather than explicit learning opens up a wide range of experimental methods that can test the generality of the cultural evolution of structure in behaviour. Recently, we have extended this method to investigate music (Ravignani, Delgado, & Kirby, 2016), and shown that six universals of rhythmic structure (Savage, Brown, Sakai, & Currie, 2015) emerge naturally from the cultural transmission of sets of drumming patterns.

## Comparative approaches to iterated learning and human uniqueness

The fact that we see systematic structure emerging even in nonlinguistic tasks opens up the obvious question about whether this cultural process is unique to humans or whether we should expect this to be observed in a wider range of species. The simplicity of the immediate imitation iterated learning experiment design described in the previous section lends itself very nicely to the construction of a nonhuman iterated learning paradigm, opening up the possibility of a comparative biology of iterated learning.

Claidiere, Smith, Kirby, and Fagot (2014) use this methodology to look at cultural evolution of sets of behaviours in a population of baboons. Rather than recalling sequences, the baboons are briefly presented with four illuminated squares in a 4 × 4 array, and then attempt to tap on the four squares that were illuminated. If they get three or four squares correct, they receive a food reward. Whatever grid pattern they produce is stored for presentation to the next baboon in the transmission chain, and a total of 50 grid patterns form the set of items that are transmitted from animal to animal.

**Fig. 10:**
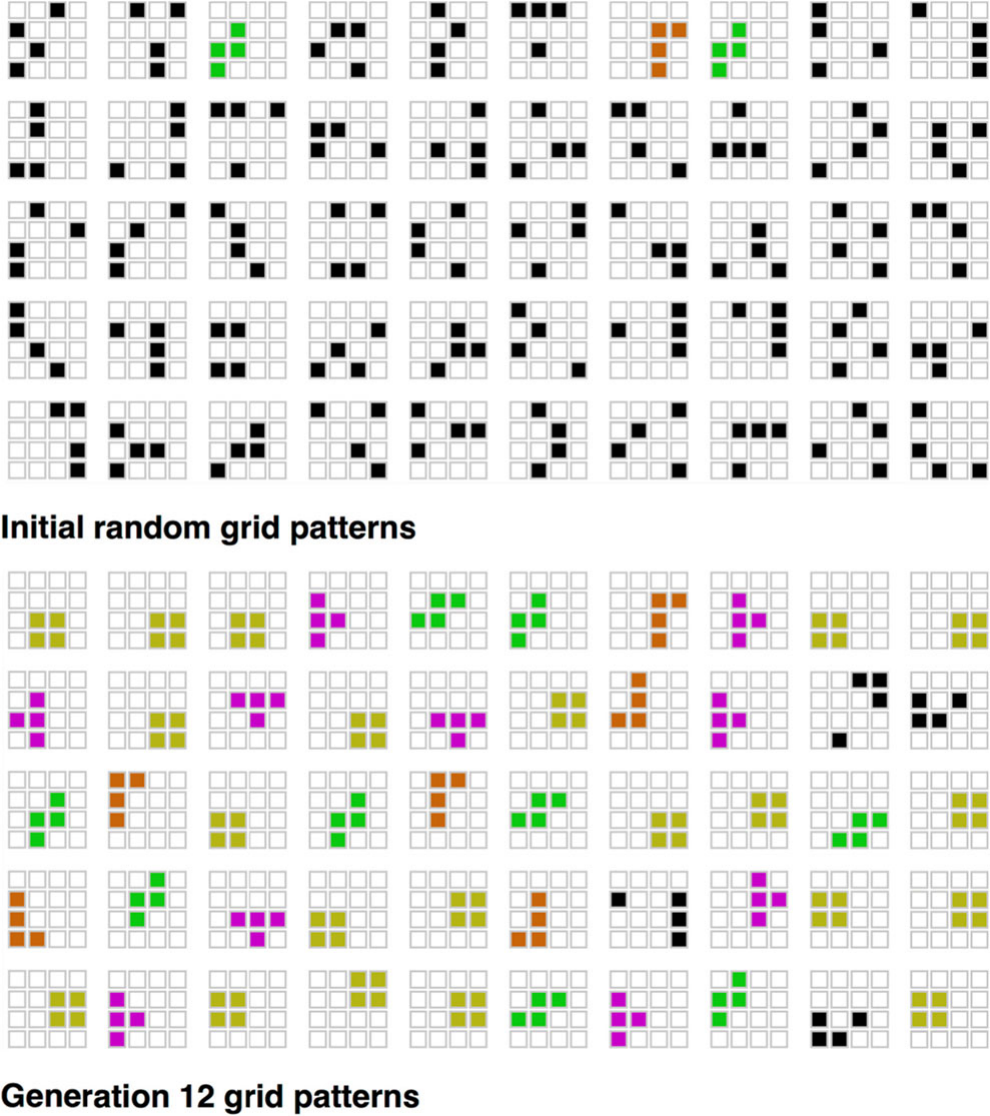
The initial and final grid patterns from one of the chains in (Claidiere et al., 2014) baboon study. The initial patterns were a set of 50 4 × 4 grids in which four cells were lit up. The baboons were rewarded for recalling the four lit squares correctly for each grid pattern, and their responses were transmitted to the next baboon in the chain. After 12 generations, the set of patterns had evolved to become systematic, with each chain having a particular distribution of statistically rare patterns known as tetrominos (highlighted in colour in the example). Importantly, the baboons did not find the tetrominos easier to copy when they occurred singly in a set of random grids. It was only when they appeared alongside other tetrominos in the set of 50 that the transmissibility advantage emerged. (Colour figure online)

Just as in the human iterated learning experiments, animals later in the chains find the task easier than those early in the chains. The set of grids gradually evolves to be easier to copy. Again, mirroring the human results, this happens by virtue of the emergence of systematicity in the set: a small number of recognisable, and hence generalisable, grid shapes emerges that are reused across the set, and different chains evolve different characteristic generalisations over the set of patterns (see Fig. 10).

This experiment demonstrates that, given the right kind of experimental setup, cumulative cultural evolution of structure in behaviour can arise in a nonhuman primate. If we cast the net a little wider, we can actually see analogs of this process in the wild. Birdsong is the most obvious candidate for a complex behaviour that is transmitted through iterated learning. In many species, male birds acquire their song through exposure to the song of the previous generation. In line with our predictions, we see systematic structure in song (Berwick, Okanoya, Beckers, & Bolhuis, 2011). Fehér, Wang, Saar, Mitra, and Tchernichovski (2009) demonstrated this experimentally in zebra finches by constructing chains of transmission in which the initial song was produced by a male bird who had been raised in acoustic isolation. Such a bird produces abnormal song which lacks a range of features that typify wild zebra finch song. Fehér et al. (2009) then allowed these isolated birds to raise a brood who learned their song from their fathers. This process was then repeated for four generations, revealing precisely how the atypical song was transmitted from one cultural generation to the next. Strikingly, the song at the end of the chains looked more like species-typical wild-type song than the abnormal isolate song at the start of the chains. Importantly, the change was gradual, with modifications to the song in the direction of wild-type structure accumulating over several generations.

The fact that the cumulative cultural evolution of structure can be observed in species other than humans raises the question of why only humans have language. If iterated learning leads straightforwardly to structure in behaviour, why don’t we see it throughout nature? As Berwick and Chomsky (2016) ask: Why only us? There must be *something* about human biology that leads to our species alone possessing language. In particular, given the results of our experiment in baboons, why don’t even our closest primate relatives exhibit either systematically structured behaviour or, indeed, cumulative culture in the wild?

I think the answer to that question comes when we look at the scaffolding we had to build into our baboon experiment design for the study to be successful. The baboons did not know that the patterns they were recreating were the product of another animal, nor that their own patterns would be transmitted to another. They were participating in social learning without being aware of it. In fact, the reason the baboons attempted to copy the grid pattern of another individual is simply because we provided a highly reliable reward structure for doing so. The behaviour of the robotic food dispenser in Claidiere and Fagot’s lab is what lead them to copy large sets of behaviours, and ultimately what lead to the cumulative cultural evolution of systematic behaviour.

Whereas the baboons had an exogenous reward mechanism that provided the platform for cultural evolution in that experiment, humans do not—at least not in most cases. Instead, uniquely among primates, we appear to have an endogenous reward mechanism for copying vast sets of behaviours. For some reason, we appear to seek out opportunities to copy not just a handful of behaviours, but whole sets of them. This drive forms part of the platform required for iterated learning.

In addition to wanting to copy, we also appear unusually predisposed to share; what Fitch (2010) calls *Mitteilungsbedürfnis.* These two predispositions—to learn signals, and to share meanings—are the two ingredients that seem to be required for our models of the emergence of compositionality through iterated learning. Our models are specifically designed to acquire signals paired with meanings, and the agents are required to produce signals for a set of meanings to transmit the behaviour they learn from one generation to the next. So, if we are looking for the unique human adaptation that gives us systematic structure in language, then perhaps we need to be looking for the biological origins of these two traits.

One approach is to look for analogous traits in other species. I will provide two suggestive examples, both involving domestication. First is the case of the so-called farm fox experiment (Belyaev, 1979; Trut, 1999). In this experiment, Siberian foxes were artificially selected purely for lack of aggression. Over the course of 30 generations, a remarkable suite of phenotypic changes took place in which the foxes took on traits typical of domesticated species, including variations in coat colour and changes in cranial size. Most strikingly, however, is that the foxes began to exhibit sensitivity to human communicative intent—a trait previously only found in dogs (Hare et al., 2005). Specifically, fox kits were able to perform as well as dog puppies in using point-and-gaze cues to find hidden food. The evidence for this kind of sensitivity in nonhuman primates is equivocal at best, and yet it is surely a prerequisite for the kind of cultural transmission required for the emergence of linguistic structure.

Second is the case of the Bengalese finch (Okanoya, 2002, 2004, 2015). These domesticated birds were bred for their plumage over the past 250 years. When compared to their wild-type predecessor, the white rumped munia, they exhibit a markedly richer song with a finite state syntax that is shaped significantly by exposure to the song of conspecifics. Crucially, the Bengalese finch song is less constrained than the munia song: Whereas munia will only acquire a very narrow range of species–specific songs, the domesticated bird’s capacity for learning is broader. It seems that here, too, a key underpinning trait required for iterated learning of language—learning signals—can be significantly shaped by domestication without specific selection on that trait itself.

Domestication appears to lift certain selection pressures off a species, triggering the evolution of analogs of the traits required for language to emerge (Deacon, 2003, 2009, 2010; Thomas, 2014). If domestication can lead to analogs of the predispositions to share and copy meaningful signals in other species, the obvious question is whether it played a role in human evolution as well. Are we a domesticated species, and did this lay the foundations for iterated learning? Thomas (2014) sets out a lengthy argument to suggest that this is indeed the case. Certainly, humans show many of the identifying features of the domesticated phenotype, marking us out as a very unusual ape. For example, reduced teeth size, decreased skeletal robustness, and cranio-facial shortening are all features of humans, and all typical of domesticated species. It is possible that changes in our environment—for example, in our feeding ecology (Hare, Wobber, & Wrangham, 2012)—led to selection for lack of aggression. This change in selective pressures on our temperament could have created in us the various features of the domestic phenotype, which we have seen elsewhere can produce the two pillars needed for the cultural evolution of communicative behaviours: learning of complex signals and sharing of communicative intentions.^7^

Once we are transmitting sets of these behaviours through iterated learning, two pressures are brought to bear on the culturally evolving system. From learning in the presence of a bottleneck comes the pressure for compressible behaviours. From the fact that the behaviours are used for discriminating meanings comes the pressure for expressivity. The languages that emerge are the product of the joint optimisation process of cultural evolution, with key structural design features of language being the inevitable outcome. The bulk of the explanatory load for this account is on cultural evolution operating with a very general simplicity bias in learning. This stands in contrast to an account that suggests many features of language arise from innate constraints on learning and are the result of natural selection for communication. Our models of gene-culture coevolution suggest that this is the least plausible explanation. We expect the language faculty to contain strong constraints only if they are domain general (e.g. arising from general principles of simplicity) and that any domain-specific constraints will be weak.

## Conclusion

Iterated learning of sets of behaviours involves repeated transmission of mental representations of those sets of behaviours through an informational bottleneck.^8^ Behaviours adapt to better pass through this bottleneck. The actual structure of the behaviours that emerges through iterated learning depends on a number of factors, not least of which is what the behaviour is used for. However, an overarching universal arising from this cultural process is that *compressible* sets of behaviours pass through the bottleneck more easily. If behaviours also need to be *expressive* then rich systematic structure appears to be the inevitable result.

This argument applies to language. The mental representations in this case are grammars, and the behaviours are utterances. The informational bottleneck is the limited data available to the child language learner. Universal properties of language emerge from simple grammars (that arise from sets of utterances evolving that are compressible) that are nevertheless expressive.

The same kind of argument could be made for other kinds of behaviours, with appropriate changes to the nature of the expressivity pressure. For example, music and dance involve sets of behaviours being transmitted culturally, with a pressure for those behaviours to be kept expressive—in the sense that there is some pressure for diversity among the set. It should be no surprise therefore that music and dance also exhibit systematic structure (Ravignani et al., 2016). We have already argued that birdsong provides a natural example of systematically structured behaviour that is shaped by iterated learning. Note again, however, that the expressivity pressure is quite different. Song does not carry semantic information and the diversity we see in song repertoires may fulfil a quite different function (e.g. signalling singer quality or good development; Nowicki, Peters, & Podos, 1998; Ritchie, Kirby, & Hawkey, 2008). As a result, it is perhaps no surprise that bird song syntax does not exhibit the kind of syntactic complexity typical of language (Berwick et al., 2011).

In this article I have given numerous examples of the way in which cultural evolution leads naturally to an increase in systematicity through cumulative evolution of compressible behaviours. The coevolutionary Bayesian models further demonstrate that the existence of cultural evolution can reduce the selection pressure on the maintenance of strong constraints on the form languages can take. Taken together, these results suggest that taking cultural evolution seriously shifts the explanatory burden for structural design features of language away from biological evolution and consequently reshapes our expectations for the nature of the language faculty.

Finally, this work suggests an approach to language evolution that takes a broad view of both the term *language* and the term *evolution*. I have argued that the unique features of language that mark it out as Nature’s most extraordinary phenotype arise from the fact that it can only persist through perpetual transformation, from internal representation (as grammars) to external manifestation (as sets of utterances) and back again, a process mediated by our language faculty. In this view it is these transformations that language goes through that matter for explaining why it is the way it is, and we do not need to take either the internal or external form to be primary. Equally, I have emphasised that “evolution” is not a process that is limited to one form of inheritance. With a trait like language, biological evolution takes place alongside individual learning and cultural transmission. We are only now beginning to understand the respective roles of these complex adaptive systems in shaping language, and the various ways they interact in doing so.

## Footnotes

1 If we consider individual features of language, we can think of this bottleneck as being more severe in some cases and less so in others. To take a very simple case, when learning the past tense of verbs, the pressure from the bottleneck will be stronger for verbs with lower frequency than those which turn up more frequently in the child’s linguistic experience. Kirby (2001) points out that this leads very naturally to the well-known fact that high-frequency verbs are more likely to be irregular in their past tense.

2 This matrix is sometimes referred to as the Q matrix in models of language evolution (Nowak, Komarova, & Niyogi, 2002).

3 Note that there is a great deal of debate about the form language universals take (see, e.g. Kirby, 1999, for review), but in general they specify a (potentially probabilistic) constraint on the ways we expect languages to vary. This is what we mean by “stationary distribution.”

4 This simplification assumes that all languages are equally effective for communication. However, the model has been extended to deal with cases where some languages confer an inherent fitness benefit (Thompson, Kirby, & Smith, 2016).

5 By this I mean a version of the model with an infinite population, with learning, culture and evolution implemented as difference equations.

6 This experiment does not include the pressure for expressivity discussed earlier. As a result, we would expect that eventually the set of sequences would become less diverse, perhaps finally collapsing to a limiting case of a single sequence repeated 60 times. Because of the huge size of the signal space, and the initial conditions of random sequences, this is likely to take many generations.

7 We can speculate whether there could have been an intermediate stage where one, but not the other trait was in place. If learning complex signals came first, then we could imagine something akin to Darwin’s (1871) theory of a protomusical ape, producing learned vocal behaviours that were not yet semantic (Fitch, 2006). If sharing of communicative intentions came first, then we may have been an ape that could improvise ostensive/inferential communication as needs demanded it, but without that communication forming a culturally transmitted conventional system (Scott-Phillips, 2014).

8 In the simulations and many of the experiments I described the bottleneck involves withholding items, forcing learners to generalise on test. However, in some experiment, such as the immediate imitation iterated learning experiments described in Section 7, the bottleneck is more indirect, arising from the limited memory of the participants in the experiment.
